# Clinical outcomes after transcatheter aortic valve replacement in cancer survivors treated with ionizing radiation

**DOI:** 10.1186/s40959-019-0044-7

**Published:** 2019-07-22

**Authors:** Nikhil Agrawal, Sharma Kattel, Sameer Waheed, Ankita Kapoor, Vasvi Singh, Ashutosh Sharma, Brian J. Page, Kristopher M. Attwood, Vijay Iyer, Saraswati Pokharel, Umesh C. Sharma

**Affiliations:** 1 Department of Medicine, Division of Cardiology, Clinical & Translational Research Center (Suite 7030), Jacob’s School of Medicine and Biomedical Sciences, Buffalo, NY USA; 2 Department of Radiology, Brigham and Women’s Hospital, Boston, MA USA; 3 Department of Biostatistics and Bioinformatics, Roswell Park Comprehensive Cancer Center, Buffalo, NY USA; 4 Department of Pathology, Division of Thoracic Pathology and Oncology, Roswell Park Comprehensive Cancer Center, Buffalo, NY USA; 5 Clinical & Translational Research Center (Suite 7030), 875 Ellicott Street, Buffalo, NY 14203 USA

**Keywords:** TAVR, Cancer survival, Radiation, And aortic stenosis

## Abstract

**Background:**

Improved cancer survival in patients treated with thoracic ionizing radiation (XRT) has resulted in unanticipated surge of aortic stenosis. Transcatheter aortic valve replacement (TAVR) has revolutionized the management of severe aortic stenosis. However, long-term clinical outcomes in radiation-exposed cohorts undergoing TAVR are unknown. We compared the all-cause mortality and major adverse cardiac events (MACE) in patients with prior chest XRT (C-XRT) undergoing TAVR.

**Methods:**

This is an observational cohort study in subjects who underwent TAVR for symptomatic severe aortic stenosis from 2012 to 2017 in a tertiary care referral center. We examined the all-cause mortality and MACE using cox proportional hazard analysis to identify the clinical predictors of survival in the cohort of patients who had a history of prior C-XRT for malignancy.

**Results:**

Of the 610 patients who underwent TAVR for symptomatic severe aortic stenosis, 75 had prior C-XRT. The majority of C-XRT patients had prior breast cancer (44%) followed by Hodgkin’s lymphoma (31%), with the median time from XRT to TAVR of 19.0 years. During a mean follow up of 17.1 months after TAVR, all-cause mortality was 17%. Those with prior C-XRT had higher all-cause mortality (XRT: 29%; non-XRT:15%, *p* < 0.01) and MACE (XRT: 57%; non-XRT: 27%, *p* < 0.001) after TAVR. Patients with prior XRT had a higher incidence of atrial fibrillation (XRT: 48%; non-XRT: 2.4%, *p* < 0.01) and high-grade heart block (XRT: 20%; non-XRT: 9.1%, *p* = 0.007) requiring pacemaker implant after TAVR. On multivariate cox proportional hazard analysis, prior XRT (HR: 2.07, *p* = 0.003), poor renal function (HR: 1.29, *p* < 0.001) and post-operative anemia requiring transfusion (HR: 1.16, p:0.001) were the strongest predictors of reduced survival.

**Conclusions:**

Cancer survivors with prior C- XRT have higher incidence of all-cause mortality and MACE after TAVR. Careful patient selection and follow-up strategies are needed to improve outcomes.

**Electronic supplementary material:**

The online version of this article (10.1186/s40959-019-0044-7) contains supplementary material, which is available to authorized users.

## Introduction

Radiation therapy is an important therapeutic modality in multiple thoracic and non-thoracic cancers [1, 2]. Chest radiation has been part of standard treatment protocol in various malignancies including lymphoma, breast, lung, and esophagus with improvement in cancer survival [3–5]. However, with increased longevity, cancer survivors face a higher rate of cardiovascular disease as a consequence of chest radiotherapy (C-XRT) [4, 6–8]. According to American Society of Clinical Oncology, cardiovascular complications tend to develop in 10–30% of patients receiving radiation therapy usually after a mean follow up of 5 to 10 years [9].

Radiation-Induced Valvular Heart Disease (RIVHD) is one of the most common late cardiac complications of C-XRT that develops in about 10% of patients undergoing C-XRT. The incidence of RIVHD increases in the second decade after radiation exposure [2, 10]. Valvular lesions are more common on the left side of the heart than the right, most commonly involving the mitral and aortic valves [11]. C-XRT leads to late scarring, fibrotic thickening, retraction and calcification of the basal and medial portions of the leaflets with sparing of the leaflet tips and commissures allowing distinction from rheumatic valve disease [2, 12, 13]. Fossa and colleagues previously reported that 39% of Hodgkin’s lymphoma survivors who underwent C-XRT developed at least moderate to severe aortic stenosis in 12 years of follow up [12]. Another retrospective analysis of C-XRT patients’ with Hodgkin’s lymphoma showed valvular disease in 6.2% of patients after an average 22 years of observation, with aortic stenosis (AS) manifesting in more than a half of these patients [14].

In patients with radiation-associated AS (RA-AS), Transcatheter Aortic Valve Replacement (TAVR) has been suggested as a safer modality compared to surgical aortic valve replacement (SAVR) as mediastinal fibrosis and aortic calcifications that develop after radiotherapy makes a surgical approach more challenging [2]. Desai and colleagues recently reported poor clinical outcomes in patients with prior C-XRT who underwent SAVR [8]. Data regarding outcomes after TAVR in patients with C-XRT are lacking, despite increasing number of patients currently being treated with transcatheter approach [15]. Therefore, we examined the long-term survival and major adverse cardiovascular events (MACE) in patients with prior C-XRT undergoing TAVR utilizing the American College of Cardiology National Cardiovascular Data Registry (STS/ACC TVT Registry).

## Methods

This is an observational cohort study in patients who underwent TAVR for symptomatic severe AS at our tertiary care referral center. All clinical procedures and protocols conformed to institutional guidelines and were approved by the Institutional review board (IRB).

### Study population and design

We studied 610 consecutive patients who underwent TAVR for symptomatic severe AS at our institute from January 2012 to September 2017. The study subjects were divided into 2 groups. The first group (XRT; *N* = 75) had prior history of C-XRT for thoracic malignancy. The second group (non-XRT; *N* = 535) had no history of C-XRT. The determination of prior C-XRT in cancer survivors was made based on chart review, or through a personal interview during their pre-TAVR evaluation visit.

### Clinical characteristics and quality of life

Baseline patient characteristics including demographics, clinical symptomatology, surgical history, radiation history, laboratory, medications use, echocardiographic and pulmonary function test were obtained at the pre-TAVR evaluation visit. Procedural and peri-procedural complications and outcomes were obtained from the procedure notes and inpatient chart review. Baseline functional status was assessed using Kansas City Quality of Life Questionnaire (KCCQ-12) at their pre-procedure clinic visit. Surgical risk was assessed using Society of Thoracic Surgery (STS) risk score.

### Pre-TAVR echocardiography

All patients underwent a comprehensive echocardiogram as part of the standard clinical diagnostic evaluation during their pre-procedure assessment for TAVR. Cardiac chamber measurements, left ventricular ejection fraction (LVEF), aortic valve area and LV stroke volume index (LV-SVI) were obtained according to current American Society of Echocardiography recommended methods [16, 17].

### Follow-up of clinical outcomes

The date of TAVR was considered as the beginning of the follow-up. Procedural and immediate postoperative data, length of intensive care unit or hospital stay and postoperative complications were retrieved from electronic medical records. All patients were routinely followed up after TAVR at 30 days and up to 1 year at our structural heart clinic. Beyond 1 year, data on all-cause mortality and MACE outcomes were obtained by reviewing shared electronic medical records with their primary care or health systems and by telephone follow-up.

The primary event was all cause mortality. Data on survival were obtained from medical record review, US Social Security Death Index or telephone follow-up. Cardiovascular mortality was defined as any death attributed to sudden cardiac arrest, myocardial infarction, arrhythmia, heart failure, or other cardiovascular causes. Major bleeding was defined as per the definitions of the International Society on Thrombosis and Hemostasis bleeding scale [18, 19].

Secondary events were composite end-point of MACE, defined as cardiovascular mortality, stroke, acute myocardial infarction (AMI) or revascularization and heart failure (HF) hospitalizations until the date of last follow up. The incidence of atrial fibrillation (AF) and atrioventricular (AV) conduction abnormalities requiring permanent pacemaker (PPM) implant were obtained through individual review of medical records, including from device clinic or through follow-up with their primary care provider. Quality of Life Questionnaire (KCCQ-12) for each patient was assessed at 30 days and at 1-year follow-up after TAVR.

### Statistical analysis

Categorical and continuous variables were expressed as percentage or frequency and mean ± standard deviation (SD) respectively where appropriate. Baseline clinical and procedural characteristics were compared between the groups using the student’s t test or the Wilcoxon rank-sum test, as appropriate, for quantitative variables; and the Pearson chi-square test for categorical variables.

All the available relevant clinical, echocardiographic, laboratory and pre/post-operative variables were used in univariate cox proportional hazard analysis to determine the association with all-cause mortality. The variables that were significant (*p* < 0.05) on univariate analysis were used to construct the multivariate cox proportional hazard model. Kaplan-Meier survival curves for all-cause mortality, composite and each component of MACE free survival were performed. A *p*-value of < 0.05 was considered statistically significant for all statistical analyses. All statistical analyses were performed using SAS statistical software, version 9.4 (SAS Institute Inc., Cary, NC).

## Results

### Comparison of baseline characteristics

#### Clinical, echocardiographic parameters and quality of life

Comparison of the baseline clinical, echocardiographic and quality of life variables are shown in Table 1. Smoking and anemia were more common and use of ACE/ARBs was less frequent in the XRT group. Both the groups had identical surgical risk (STS score) and functional status (KCCQ-12) scores at baseline.

**Table 1:** Baseline characteristics of the study population

Variables^a^	All subjects (*n* = 610)	Non- XRT Group (*n* = 535)	C-XRT Group (*n* = 75)	*P* value
Clinical, demographic, and symptom variables				
Age (years)	82.00 ± 7.99	82.67 ± 7.98	81.64 ± 7.81	0.21
Gender (M/F)	320/290	291/244	29/46	0.013
Body Mass Index, kg/m^2^	27.99 ± 6.02	28.11 ± 5.98	27.14 ± 6.32	0.10
Race				0.38
White	587 (96.4)	516 (96.6)	71 (94.7)	
Blacks	16 (2.6)	12 (2.2)	4 (5.3)	
Hispanics	3 (0.5)	3 (0.6)		
Others	3 (0.5)	3 (0.6)		
Hypertension	542 (88.8)	476 (88.9)	66 (88)	0.84
Diabetes mellitus	205 (33.60)	176 (32.5)	31 (41.3)	0.15
Hyperlipidemia	355 (58.19)	307 (57.3)	48 (64)	0.31
Smoking history	304 (49.83)	251 (46.9)	53 (70.6)	< 0.0001
Prior stroke	63 (10.33)	53 (9.9)	10 (13.33)	0.41
COPD	263 (43.11)	229 (42.8)	34 (45.33)	0.7
CAD	357 (58.52)	307 (57.3)	50 (66.67)	0.13
3-Vessel CAD	170 (27.9)	147 (27.5)	23 (30.7)	0.58
End-stage renal disease	31 (5.08)	26 (4.8)	5 (6.67)	0.57
Atrial fibrillation	249 (40.81)	223 (41.6)	26 (34.6)	0.26
PAD	224 (36.72)	201 (37.57)	23 (30.6)	0.3
Prior CABG	192 (31.47)	166 (31.0)	26 (34.67)	0.51
Pacemaker Implant History	100 (16.39)	82 (15.33)	18 (24.00)	0.06
ICM (EF < 50% with CAD)	111 (18.19)	97 (18.1)	14 (18.67)	0.87
NYHA Class				0.36
I	4 (0.7)	4 (0.7)		
II	76 (12.5)	65 (12.1)	11 (14.7)	
III	462 (75.7)	410 (76.6)	52 (69.3)	
IV	68 (11.1)	56 (10.5)	12 (16.0)	
Syncope	38 (6.2)	33 (6.17)	5 (6.67)	0.8
Angina	194 (31.8)	172 (32.15)	22 (29.33)	0.69
Dyspnea on exertion	555 (91)	487 (91.03)	68 (90.67)	0.83
FEV1 (% of predicted)	75.14 ± 24.6	75.16 ± 24.81	75.02 ± 23.73	0.96
STS score, Mean	9.03 ± 5.4	9.02 ± 5.41	9.11 ± 5.06	0.7
STS Score, Median (Interquartile)	8.1 (5.3–11)	8.1 (5.3–11)	8.1 (5.4–11)	
KCCQ12 Index	36.4 ± 19.6	36.80 ± 19.78	33.72 ± 18.2	0.2
Laboratory data				
Creatinine, mg/dl	1.4 ± 1.1	1.38 ± 1.12	1.39 ± 1.10	0.92
Glomerular filtration rate, mL/min per 1.73m^2^	53.6 ± 22.3	53.68 ± 22.45	52.79 ± 21.4	0.74
Hemoglobin, mg/dl	12 ± 1.8	12.07 ± 1.80	11.60 ± 1.49	0.03
Medications				
Aspirin	402 (66)	354 (66.1)	48 (73.8)	0.26
Beta-blockers	491 (80.5)	433 (80.9)	58 (81.6)	1
ACE-I/ARBs	215 (35.2)	200 (37.3)	15 (23.0)	0.02
Statins	356 (58.4)	307 (75.3)	49 (65.3)	0.21
Echo Parameters				
LVEF	54.6 ± 13	54.46 ± 13.1	55.65 ± 12.40	0.45
AVA (cm2)	0.65 ± 0.2	0.65 ± 0.2	0.63 ± 0.2	0.36
Mean AV gradient (mm Hg)	41.14 ± 15.28	40.87 ± 15.48	43.06 ± 13.67	0.24
Peak AV gradient (mm Hg)	67.04 ± 24.72	66.90 ± 25.02	68.05 ± 22.61	0.7
LV-SVI (ml/m2)	37.14 ± 13.50	36.72 ± 13.49	40.11 ± 13.30	0.04
Abnormal LV-SVI (< 35 mL/m2)	294 (48.2)	271 (50.65)	23 (30.67)	0.001
RVSP (mm Hg)	47.24 ± 14	47.11 ± 14.13	48.18 ± 13.07	0.53
Moderate-Severe AR	99 (16.22)	85 (15.89)	14 (18.66)	0.52
Moderate-Severe MR	127 (20.81)	106 (19.81)	21 (28.00)	0.21
Moderate-Severe TR	110 (18.03)	95 (17.76)	15 (20.00)	0.48
Moderate-Severe MS	45 (7.37)	34 (6.36)	11 (14.67)	0.01

C-XRT indicates mediastinal radiation therapy

*CAD* coronary artery disease, *COPD* chronic obstructive pulmonary disease, *PAD* periphery artery disease, *CABG* coronary artery bypass grafting, *ICM* ischemic cardiomyopathy, *FEV1* forced expiratory volume at 1 s, *STS* Society of Thoracic Surgeons, *KCCQ12* Kansas City Cardiomyopathy Questionnaire, *ACE-I/ARBs* Angiotensin-converting enzyme inhibitors/ Angiotensin II receptor, *LVEF* left ventricular ejection fraction, *AVA* aortic valve area, *AV* aortic valve, *LV-SVI* left ventricular stroke volume index, *RVSP* right ventricular systolic pressure, *AR* aortic regurgitation, *MR* mitral regurgitation, *TR* tricuspid regurgitation, *MS* mitral stenosis

^a^Values are expressed as number (percentage) or mean ± SD

#### Natural history and presence of multivalvular lesions

The median time from C-XRT to TAVR was 19.0 years (Mean 20.1 ± 4.9 yrs.). Of the 75 symptomatic severe aortic stenosis patients with prior C-XRT who underwent TAVR, breast cancer (44%) was the commonest reason for C-XRT followed by Hodgkin’s lymphoma (31%), lung cancer (15%), non-Hodgkin’s lymphoma (7%) and others (3%). Among breast cancer survivors who had C-XRT, 64% had left sided malignancy.

Moderate to severe aortic, mitral and tricuspid regurgitation was seen in 16.2, 20.8 and 18% of the total study subjects, respectively. The incidence of moderate-severe mitral stenosis was disproportionately higher in the XRT group.

#### Age and sex disparities

Females were disproportionally more frequent among the cancer survivors with prior C-XRT (XRT: 61%, non-XRT: 45%, *p* = 0.01), which likely reflects the higher proportion of breast cancer survivors in the XRT group. The male population was slightly younger than females at the time of TAVR (mean age, male/female: 81.3 ± 8.2 vs 82.8 ± 7.6, *p* = 0.02). Mean STS score was higher in females (male/female: 8.2 ± 4.6 vs 9.9 ± 5.9, *p* < 0.0001).

The incidence of AF and AV block requiring PPM were more common in females but there were no differences in all-cause mortality, MACE, major bleeding, or length of hospital stay among male or female patients.

Females subjects had lower KCCQ-12 scores at 30 days and 1-year follow-up compared to male counterparts (male/female, mean score at 30 days: 80.88 ± 15.88 vs. 77.46 ± 18.20, *p* = 0.01; at 1 year: 84.51 ± 12.14 vs. 81.44 ± 14.61, *p* = 0.008) though there was no difference in their baseline score.

In a subgroup analysis of all females who underwent TAVR (*N* = 290, XRT = 46; non-XRT = 244), females who had C-XRT were slightly younger (mean age, XRT vs. without XRT: 80.8 ± 8.4 vs. 83.2 ± 7.5, *p* = 0.04).

### Comparison of Peri-procedural events

Perioperative and post-operative characteristics are shown in Table 2. There were no differences in intravascular access, procedure duration or type of valves used among the groups.

**Table 2:** Perioperative and Postoperative Characteristics of the Study Population

Variable^a^	All subjects (*n* = 610)	Non- XRT Group (*n* = 535)	C-XRT Group (*n* = 75)	*P* value
TAVR Access/duration				
Femoral Access	535 (87.7)	472 (88.22)	63 (84.0)	0.34
Median Procedure duration (hrs:mins/ interquartile)	2:17 (1:40–2:48)	2:16 (1:04–3:28)	2:23 (1:29–3:07)	0.38
Valve types				
Edwards Sapien	500 (81.9)	438 (81.87)	62 (82.67)	0.86
Core Valve	110 (18.1)	97 (18.13)	13 (17.33)	
Post-procedure parameters				
Mod-severe Paravalvular leak	9 (1.48)	8 (1.5)	1 (1.35)	0.91
Post-procedure Hb	9.66 ± 1.75	9.73 ± 1.76	9.12 ± 1.6	0.004
Post-procedure Creatinine	1.52 ± 1.34	1.50 ± 1.28	1.64 ± 1.7	0.41
Creatinine at discharge	1.3 ± 1.1	1.3 ± 1.1	1.28 ± 1.1	0.73
Post TAVR LVEF	55.05 ± 12.4	54.72 ± 12.74	57.4 ± 9.8	0.081
Post TAVR AVA, cm^2^	1.90 ± 0.49	1.91 ± 0.49	1.87 ± 0.5	0.53
Post TAVR Peak gradient, mm Hg	7.93 ± 4.64	7.94 ± 4.59	7.87 ± 5	0.9
Post TAVR mean gradient, mm Hg	3.62 ± 2.28	3.62 ± 2.23	3.64 ± 2.6	0.94

C-XRT indicates mediastinal radiation exposure

*Hb* hemoglobin, *LVEF* left ventricular ejection fraction, *AVA* aortic valve area

^a^Values are expressed as number (percentage) or mean ± SD

The overall incidence of moderate to severe paravalvular leak post-TAVR was less than 1.5% in the entire study subjects and there were no significant differences among the groups.

### Post-TAVR outcomes

The short and long-term events and quality of life measures after TAVR are shown in Table 3.

**Table 3:** Short and long-term outcomes post-TAVR

Outcomes^a^	All Subjects (*n* = 610)	Non- XRT Group (*n* = 535)	C-XRT Group (*n* = 75)	*P* value
Early Events				
ICU LOS (hrs)	41.9 ± 43.1	41.50 ± 43.19	44.67 ± 43.00	0.55
Hospital LOS (days)	5.2 ± 6.5	5.32 ± 6.84	4.52 ± 3.64	0.31
In Hospital AMI	1 (0.16)	1 (0.19)	0 (0)	1.0
In Hospital AF	49 (8.03)	13 (2.43)	36 (48.00)	< 0.0001
In Hospital stroke	9 (1.48)	5 (0.93)	4 (5.33)	0.01
In Hospital cardiac arrest	20 (3.28)	15 (2.80)	5 (6.67)	0.08
In Hospital Mortality	17 (2.79)	12 (2.24)	5 (6.67)	0.04
30-day Mortality	17 (2.79)	12 (2.24)	5 (6.67)	0.04
Major bleed	63 (10.33)	52 (9.72)	11 (14.67)	0.22
Long-term Events				
AMI and/or urgent PCI	35 (5.74)	31 (5.79)	4 (5.33)	1.00
Stroke/TIA	36 (5.90)	27 (5.05)	9 (12.00)	0.03
HF admission	113 (18.52)	90 (16.82)	23 (30.67)	0.006
PPM implants	64 (10.49)	49 (9.16)	15 (20.0)	0.007
CV Mortality	50 (48.54)	36 (44.44)	14 (63.64)	0.14
All-Cause Mortality	103 (16.89)	81 (15.14)	22 (29.33)	0.004
MACE	185 (30.33)	144 (26.92)	41 (54.67)	< 0.0001
Quality of Life measures				
Home Disposition post TAVR	513 (84.10)	451 (84.30)	62 (82.67)	0.73
KCCQ12 at 30 days	79.23 ± 17.11	79.27 ± 16.98	78.96 ± 18.20	0.88
KCCQ12 at 1 year	83.02 ± 13.47	82.86 ± 13.55	84.29 ± 12.81	0.44

C-XRT indicates mediastinal radiation therapy

*ICU* intensive care unit, *LOS* length of stay, *AF* atrial Fibrillation, *AMI* acute myocardial infarction, *PCI* percutaneous coronary intervention, *TIA* transient ischemic attack, *HF* heart failure, *PPM* permanent pacemaker, *CV* cardiovascular, *MACE* major cardiovascular adverse event (CV death/MI/Stroke/HF), *KCCQ12* Kansas City Cardiomyopathy Questionnaire

^a^Values are expressed as number (percentage) or mean ± SD

#### Short-term outcomes

The overall in-hospital incidences for AMI, AF, stroke and all-cause mortality were 0.5, 8, 1.5, and 2.8%, respectively. XRT group had higher incidence of in-hospital AF, stroke and all-cause mortality but no difference in 30 days mortality or major bleeding among the groups.

#### Long-term outcomes

During a mean follow up of 17.1 months (median: 13 months) post-TAVR, the all-cause mortality was 17% in the study population. Similarly, the incidence of MACE was 30% in the entire study population. There were significantly higher rates of mortality and MACE in the XRT group (XRT/non-XRT groups: Death, 29% vs 15%, *p* = 0.004; MACE, 54% vs 27%, *p* < 0.0001, respectively). The overall incidence of high-grade AV block requiring PPM implantation was 10.5% in all the study subjects and was disproportionally higher in XRT group (XRT/non-XRT group: 19% vs 9%, *p* = 0.001).

**Fig. 1:**
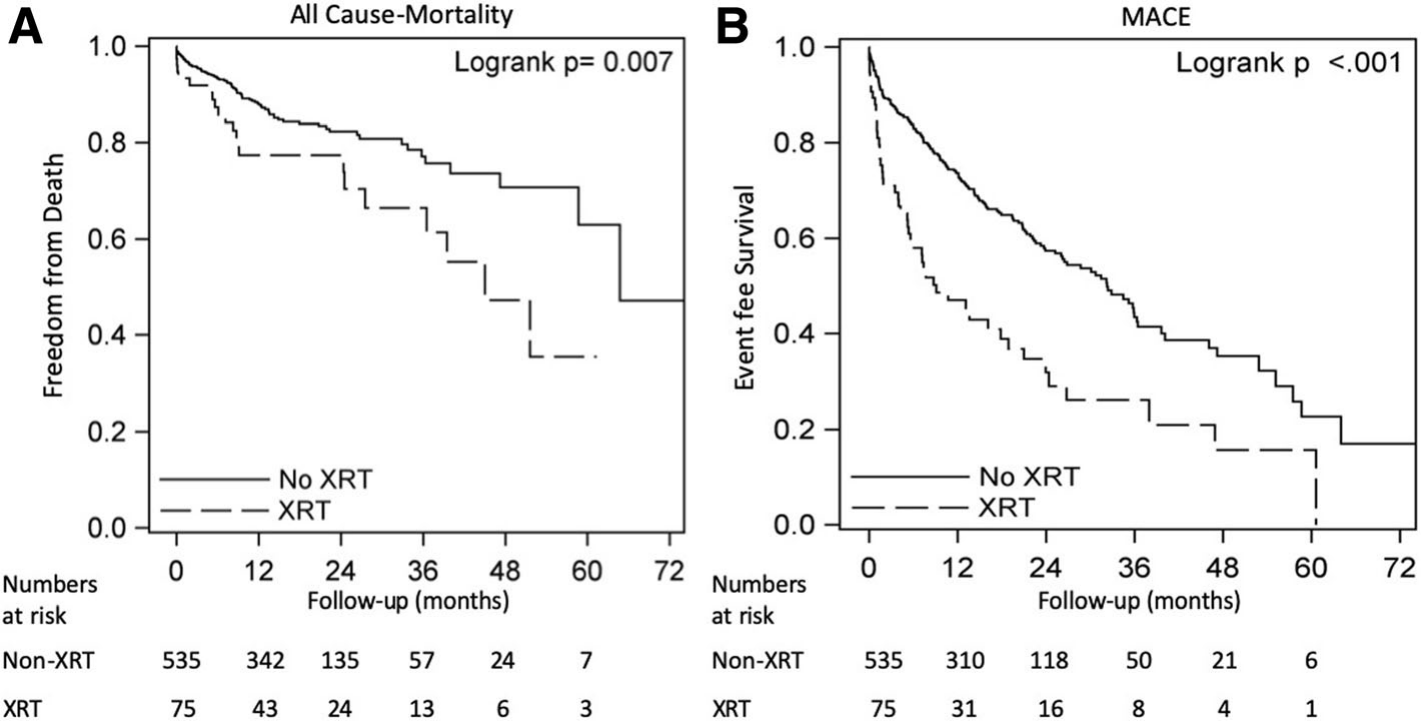
**a-b** Kaplan-Meier survival curves for all-cause mortality and MACE (major adverse cardiovascular events) in the entire study population separated into 2 subgroups: chest radiotherapy (XRT group) versus comparison group (Non-XRT)

**Fig. 2:**
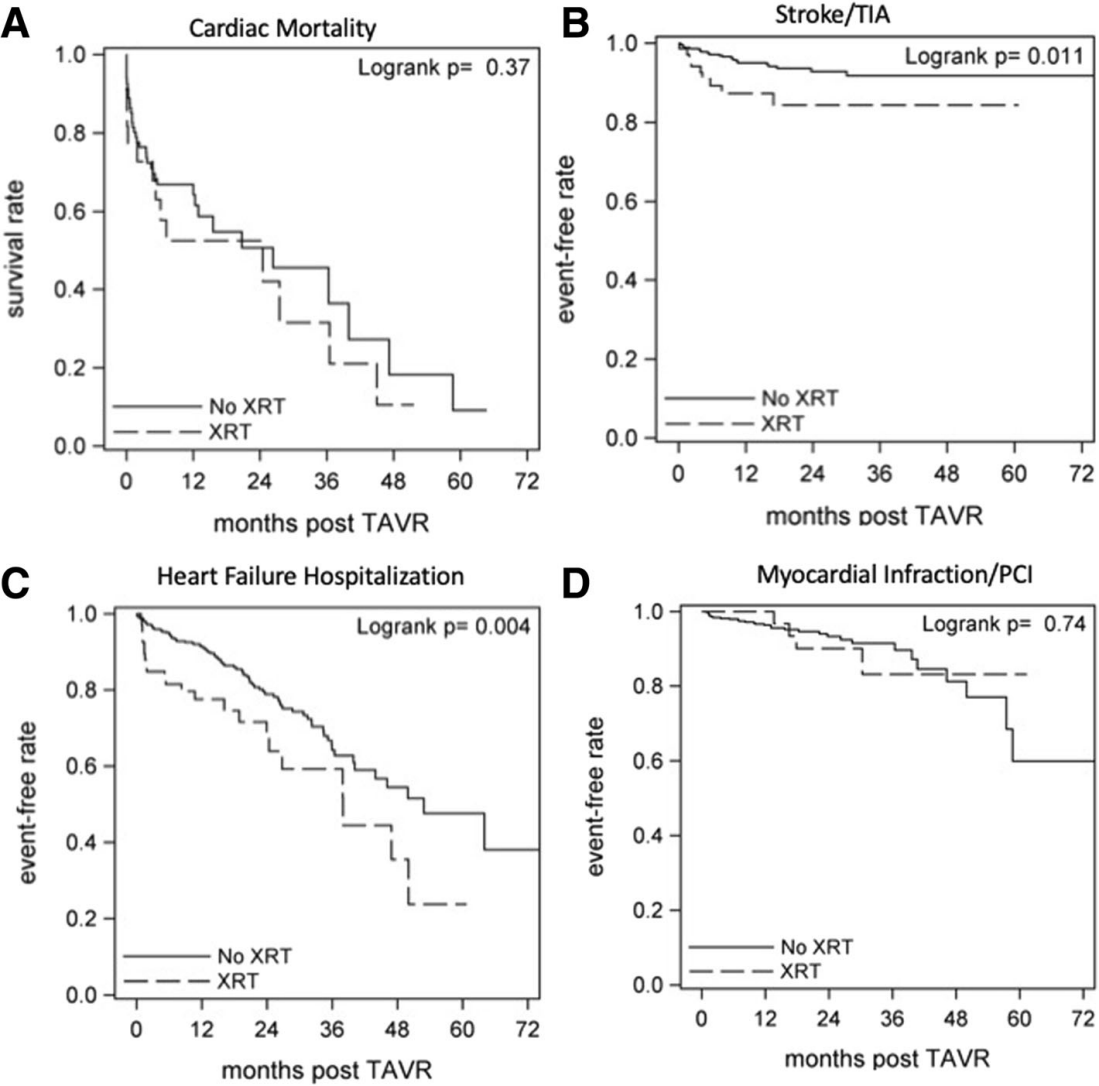
**a-d** Kaplan-Meier curves for **a** cardiac mortality, **b** stroke/TIA, **c** heart failure hospitalization and **d**myocardial infraction/PCI in the entire study population separated into 2 subgroups: chest radiotherapy (XRT group) versus comparison (Non-XRT group)

Time to event survival analysis by Kaplan-Meier analysis showed reduced survival and increased incidence of MACE in the XRT group as shown in Fig. 1a-b. Similarly, Kaplan-Meier analysis showed increased incidence of HF and stroke but not for CV mortality and AMI or urgent revascularization in the XRT group as shown in Fig. 2a-d.

The results of the univariate and multivariate cox proportional hazard analysis for all-cause mortality are shown in Table 4. Prior C-XRT exposure (HR: 2.07; 95% CI:1.24–3.31, *p* = 0.005), poor renal function post TAVR (HR: 1.43, 95% CI:1.11–1.85, *p* = 0.004) and post-operative anemia requiring transfusion (HR: 1.17; 95% CI: 1.05–1.30, *p* = 0.003) were the strongest predictors of reduced survival.

**Table 4:** Univariate and multivariable cox proportional hazard analysis for all-cause mortality

Variables	Univariate analysis		Multivariate analysis	
	Hazard Ratio (95% CI)	*p* value	Hazard Ratio (95% CI)	*p* value
Clinical variables				
Prior XRT	1.94 (1.18–3.06)	0.009	2.07 (1.24–3.31)	0.005
STS score (per unit change)	1.04 (1.01–1.07)	0.001	1.00 (0.96–1.03)	0.93
Age (per unit change)	0.99 (0.97–1.02)	0.7		
Gender (Male versus Female)	1.2 (0.81–1.78)	0.35		
BMI (kg/m^2^, per unit change)	0.99 (0.96–1.03)	0.9		
Hypertension	1.21 (0.62–2.72)	0.58		
Diabetes mellitus	1.00 (0.66–1.50)	0.96		
Hyperlipidemia	1.54 (1.02–2.37)	0.03	1.48 (0.96–2.31)	0.07
Smoking	1.37 (0.92–2.04)	0.11		
Prior Stroke	1.60 (0.98–2.51)	0.05		
COPD	1.49 (1.01–2.20)	0.04	1.21 (0.74–2.00)	0.43
CAD	1.29 (0.86–1.97)	0.21		
3 Vessel CAD	0.85 (0.53–1.32)	0.49		
End stage renal disease	3.03 (1.61–5.23)	0.001	0.96 (0.28–2.92)	0.94
Peripheral artery disease	1.24 (0.82–1.84)	0.29		
Atrial Fibrillation	1.30 (0.87–1.92)	0.18		
Prior Cardiac surgery	0.90 (0.59–1.36)	0.65		
ICM (EF < 50% and CAD)	1.25 (0.73–1.89)	0.44		
Previous PCI	1.03 (0.62–1.64)	0.88		
Prior Pacemaker	1.24 (0.74–1.99)	0.38		
Angina	0.97 (0.90–1.46)	0.9		
Syncope	1.21 (0.53–2.36)	0.61		
Dyspnea on exertion	1.37 (0.70–3.10)	0.36		
Pre-TAVR KCCQ12 score (per unit change)	0.37 (0.12–1.06)	0.06		
Lab /Echo Variable				
Pre-TAVR Hb (gm, per unit change)	0.79 (0.70–0.89)	0.0002	090 (0.78–1.04)	0.17
Creatinine (per unit change)	1.22 (1.09–1.33)	0.0006	0.87 (0.66–1.13)	0.32
eGFR (per unit change)	0.99 (0.98–1.00)	0.13		
FEV1 (per unit change)	0.98 (0.98–0.99)	0.01	0.99 (0.98–1.00)	0.43
LV EF (%, per unit change)	0.99 (0.98–1.01)	0.59		
AR (per level increase in severity)	1.26 (0.71–2.24)	0.06		
Presence of MS	1.80 (0.96–3.12)	0.06		
MR (per level increase in severity)	1.32 (0.69–2.51)	0.33		
TR (per level increase in severity)	0.81 (0.41–1.58)	0.36		
AVA (cm2, per unit change)	1.38 (0.47–3.87)	0.54		
Mean AV gradient (mmHg, per unit change)	0.99 (0.98–1.00)	0.43		
Peak AV gradient (mmHg, per unit change)	0.99 (0.99–1.00)	0.88		
Reduced LV-SVI	1.13 (0.76–1.67)	0.51		
RVSP (mmHg, per unit change)	1.01 (1.00–1.02)	0.01	1.01 (0.99–1.02)	0.05
Medications				
Aspirin	1.15 (0.76–1.78)	0.5		
Beta-Blocker	1.37 (0.79–2.58)	0.27		
Statins	1.52 (0.99–2.32)	0.05		
ACE/ARBs	1.05 (0.69–1.57)	0.81		
Operative and Post-Operative variables				
Operative time (in hours, per unit change)	1.00 (0.99–1.00)	0.09		
TVT Access (Femoral vs others)	1.07 (0.63–1.94)	0.8		
Type of Valve (Edward Sapiens vs Core valve)	0.77 (0.48–1.29)	0.31		
Para-valvular leak (mod-severe)	0.58 (0.09–2.01)	0.18		
Post Op Atrial fibrillation	1.55 (0.82–2.68)	0.16		
Creatinine at discharge (mg, per unit change)	1.33 (1.19–1.47)	< 0.001	1.43 (1.11–1.85)	0.004
Post procedure Hb (gm, per unit change)	0.82 (0.72–0.92)	0.001	1.03 (0.87–1.23)	0.66
Per units’ blood transfused	1.22 (1.12–1.32)	< 0.0001	1.17 (1.05–1.30)	0.003
Post-LVEF (%, per unit change)	0.99 (0.97–1.00)	0.29		

Chi-square for the overall multivariate model was 54.82, *p* < 0.0001. XRT indicates mediastinal radiation exposure

*STS* Society of Thoracic Surgeons, *BMI* body mass index, *CAD* coronary artery disease, *COPD* chronic obstructive pulmonary disease, *ICM* ischemic cardiomyopathy, *PCI* percutaneous coronary intervention, *Hb* hemoglobin, *LVEF* left ventricular ejection fraction, *AVA* aortic valve area, *AV* aortic valve, *LV-SVI* left ventricular stroke volume index, *RVSP* right ventricular systolic pressure, *AR* aortic regurgitation, *MR* mitral regurgitation, *TR* tricuspid regurgitation, *MS* mitral stenosis, *FEV1* forced expiratory volume at 1 s, *KCCQ12* Kansas City Cardiomyopathy Questionnaire

#### Quality of life outcomes

Nearly 84% of the subjects were able to leave the hospital on average 6 days after TAVR. There was an increased trend toward a longer in-hospital stay in XRT group, however this didn’t reach statistical significance. Functional status as measured by KCCQ-12 at baseline, 30 days and 1 year after TAVR were similar among groups as shown in Additional file 1: Figure S1. When compared in all subjects, those who were surviving had higher recovery of KCCQ-12 at 30 days, however no difference at 1 year as shown in Additional file 2: Figure S2.

### Subgroup analysis within the XRT group

#### Age and sex disparities in XRT group

In subgroup analysis of the XRT group (*N* = 75), there was no difference in the mean STS score among male or female subjects. Angina was more common in female subjects (male/female: 13.7% vs. 39.13%, *p* = 0.02). Overall, 50% of the patients had prior CAD with a higher incidence in male patients (male vs. female: 82.7% vs. 56.5%, *p* = 0.02). All-cause mortality was higher in males compared to females (male/female:51.7% vs 15.22%, *p* < 0.001), however there was no differences in 30-day mortality, MACE, KCCQ-12 among male or female subjects. After TAVR, there was an average improvement of KCCQ-12 scores by 44.5 ± 21.02 and 48.95 ± 19.87 points at 30 days and 1 year, respectively, which was similar compared to all subjects in the study.

#### Clinical outcomes in relation to cancer types

In cancer survivors, the all-cause mortality differed according to the cancer types, which has been summarized in the Additional file 3: Table S1. Similarly, the MACE outcomes differed according to the cancer types and were highest among Hodgkin’s lymphoma patients. The incidence of stroke was also highest among Hodgkin’s lymphoma followed by breast cancer, and none in other cancer survivors during the follow-up period.

## Discussion

Our study demonstrates that patients who underwent TAVR with prior C-XRT for thoracic cancers have poor survival compared to the those who had undergone same procedure. Furthermore, those who had prior C-XRT had less than 50% freedom from MACE during average of 17.1 months follow-up. This is the first study evaluating the long-term outcome of TAVR in thoracic cancer survivors with prior history of C-XRT.

### Prognostic impact of prior chest radiation on survival

Our data demonstrated that the patients with prior C-XRT had nearly 2-fold increase in mortality after the median follow-up duration of 17 months after TAVR. The mortality differences between the XRT and control groups were discernible at the early in-hospital period and became more prominent with increasing follow-up interval. Our multivariate analysis revealed prior C-XRT, post-operative anemia requiring blood transfusion and poor renal function as the significant predictors of reduced survival. Larger multicenter studies on the long-term outcomes of RIVHD are limited. Based on a recent study in patients with SAVR and CABG, presence of prior C-XRT was associated with worse longer-term survival [8]. Other smaller studies in prior C-XRT have also reported the presence of constrictive pericarditis, reduced pre-operative LVEF, concomitant pulmonary fibrosis, longer cardiopulmonary bypass time and hostile chest environment (radiation induced fibrosis/adhesions, and presence of multiple cardiac lesions) to be strongly associated with increased mortality [20–23]. In our study, we found no significant differences in pre-operative LVEF and FEV1 in patients with prior C-XRT and hostile chest environment likely have less impact in these patients since TAVR involves percutaneous approaches. Besides, there was higher incidence of HF, AF, stroke and AV conduction abnormalities requiring PPM in the XRT subsets, which might also have contributed to the increased mortality. These data are important for prior adjudication and counseling of patients prone to develop such complications.

### Incidence of atrial fibrillation, stroke, heart failure and conduction abnormalities

We noted higher incidence of AF in XRT cohorts after TAVR. Prior study from Siregar and associates showed increased incidence of AF after cardiac surgery in Hodgkin’s lymphoma survivors with history of C-XRT [24]. Other studies have also demonstrated a higher prevalence of AF in patients with history of cancer. One plausible explanation for this observation is the presence of shared risk factors for AF and cancer including age, higher body mass index, hypertension and history of smoking [25]. There is evidence that radiation induces a low-grade inflammation in the myocardium which can lead to progressive interstitial fibrosis [26]. Presence of inflammation and fibrosis in the atrial tissues can increase the propensity to develop AF. Ascertainment of the mechanisms of increased incidence of AF in these patients will probably need cardiac MRI with comprehensive tissue characterization or histological analysis of the affected cardiac tissue.

Additionally, patients with prior C-XRT showed nearly double the incidence of stroke post-TAVR. The incidence of stroke in our patients without prior C-XRT was identical to previously reported incidence at 3–6% [27]. Patients with prior C-XRT also had higher incidence of AF, which might have contributed to the increased incidence of stroke, but it’s also plausible that these patients have higher incidence of atherosclerosis and aortic calcifications that are known to increase the propensity for stroke with percutaneous vessel manipulation during TAVR [14, 28, 29]. With increasing use of distal protective devices during TAVR, the incidence of stroke, particularly related to atheroma breakdown, is expected to decline [30]. Moreover, patients with Hodgkin’s lymphoma had higher incidence of stroke compared to other cancer subtypes which may relate to increased risk of atherosclerosis from higher dose of mediastinal radiation on the large arteries. Future prospective studies will need to address whether oral anticoagulation is beneficial in these patients.

The incidence of HF hospitalization was almost double in the XRT group (nearly 31%) despite no differences in their baseline LVEF. Prior studies have demonstrated that nearly a quarter of the patients return to hospital within a year due to HF post-TAVR [31]. A seminal study by Durand and associates reported that pre-TAVR low aortic mean gradient, left atrial dilation, post-procedure anemia requiring blood transfusion and persistent severe pulmonary hypertension post-TAVR were associated with increased incidence of HF hospitalization [31]. In our XRT group, aortic mean gradients before and after TAVR, and right ventricular systolic pressure at baseline were similar compared to non-XRT group. However, the incidence of anemia and need for blood transfusion was higher in XRT group which is consistent with earlier findings and might have played a contributory role for cardiac decompensation. It is also important to state the role of diastolic function in HF patients with C-XRT since it is known to induce myocardial fibrosis perpetuating diastolic dysfunction, which was not completely addressed in our study [26, 32].

The incidence of PPM implant in our patients undergoing TAVR was ~ 10%. Notably, patients with C-XRT had double the incidence (~ 20%) of conduction abnormalities requiring PPM. While the exact mechanisms for these conduction abnormalities are not well understood, these are likely contributed by microvascular damage, ischemia of the conducting myocytes or direct injury of the sinoatrial node, atrioventricular node and conducting myocytes [32]. This is an important finding since TAVR procedure in itself has higher incidence of high-grade conduction abnormalities compared to SAVR with need for PPM approaching up to 25% in some studies. In patients with prior C-XRT, a close monitoring is needed to detect and treat these life-threating conduction diseases [33].

### Gender disparity within C-XRT TAVR

We noted higher incidence of all-cause mortality in male counterparts compared to females who had prior C-XRT but no significant differences in MACE or cardiac mortality. Though males had higher prevalence of underlying CAD and lower pulmonary functional capacity at baseline, females had more anginal symptoms compared to male counterparts who had prior chest XRT. Previous studies by Chandrasekhar et al. and Hayashida et al. showed that females had better survival at 1 year after TAVR compared to male counterparts [34, 35]. Thus, it is important to note that outcomes of TAVR based on gender analysis is similar in patients with prior C-XRT versus general population undergoing TAVR and male had overall worse outcomes. The effects of chest radiation on gender in these subsets of patients are unknown and warrant further studies.

### Quality of life

The overall baseline functional status of all the patients was poor based on KCCQ-12 score categorization, with a trend towards a very poor category in patients with prior C-XRT [36]. The overall functional status seemed similar to previously reported data from the TVT registry [37]. On average, post TAVR, there were similar improvements in both the groups by more than average of 40 points in the KCCQ12 scores at 30 days and at 1-year after TAVR which are slightly higher than previously reported in clinical trials or data from TVT registry [36–38]. Our study also shows that patients with lower pre-procedure and smaller improvement of the KCCQ-12 score post-TAVR are likely to have higher mortality. This finding can further guide the clinicians in selecting patients who can benefit from TAVR and help prognosticating the outcomes after TAVR.

### Limitations

We acknowledge the following limitations. First, our study is an observational cohort study and lacks the inherent strength of a randomized controlled trial. However, this study provides important insight into the natural history of TAVR for symptomatic severe aortic stenosis patients with prior history of C-XRT. Second, there remains the concern for an adequate capture of clinical outcomes mainly resulting from lack of formal follow-up beyond 12-months. For patients who did not follow up clinically in our health-care system, we obtained their data by shared clinical chart review and standardized telephone interview. Besides, the trends of primary and secondary events separated significantly early between the two groups and continued to widen over the duration of follow-up which suggests a potentially even greater magnitude of difference over time. Third, our study was not able to specify the radiation dose and adjuvant chemotherapy regimens which might have impacted the cardiovascular outcomes. We know that anthracyclines can cause cardiotoxicity leading to cardiomyopathy and heart failure, but antracyclines are not known to cause valvular stenosis. The effect of additional adjuvant regimens in patients with prior C-XRT undergoing TAVR is undefined and needs further study [39] [40].,

## Conclusions

Based on this observational study, we conclude that patients with prior history of chest radiation treatment are at higher risk of adverse cardiovascular events and poor survival after TAVR. These patients may require more robust screening, follow up and clinical vigilance as they tend to do worse than general population undergoing TAVR. These data will also help in pre-procedure counselling of these patients.

## Additional files

Additional file 1: **Figure S1.** Graph showing KCCQ12 score at pre TAVR, 1 month and one-year post TAVR in the entire study population and separated into 2 subgroups: chest radiotherapy (XRT group) versus comparison (Non-XRT group). (JPG 79 kb)

Additional file 2: **Figure S2.** Graph showing KCCQ12 score at pre TAVR, 1 month and one-year post TAVR in the entire study population and separated into 2 subgroups: surviving vs non-surviving groups). * denotes statistically significant differences among the groups. (JPG 87 kb)

Additional file 3: **Table S1.** Clinical Outcomes in Relation to Types of Malignancies Treated with Ionizing Radiation. (DOCX 16 kb)

## Data Availability

The published data will be made available upon satisfactory written request.

## Abbreviations

AF: Atrial fibrillation
AMI: Acute myocardial infarction
AV: Atrioventricular
HF: Heart Failure
KCCQ-12: Quality of Life Questionnaire
MACE: Major adverse cardiac events
PPM: Permanent pacemaker
RIVHD: Radiation-Induced Valvular Heart Disease
XRT: Thoracic ionizing radiation
