# Supplementary figures and images for: Clinical outcomes after transcatheter aortic valve replacement in cancer survivors treated with ionizing radiation
Source: Cardiooncology. 2019 Jul 22;5:8. doi: 10.1186/s40959-019-0044-7 (PMC6897372; doi:10.1186/s40959-019-0044-7)

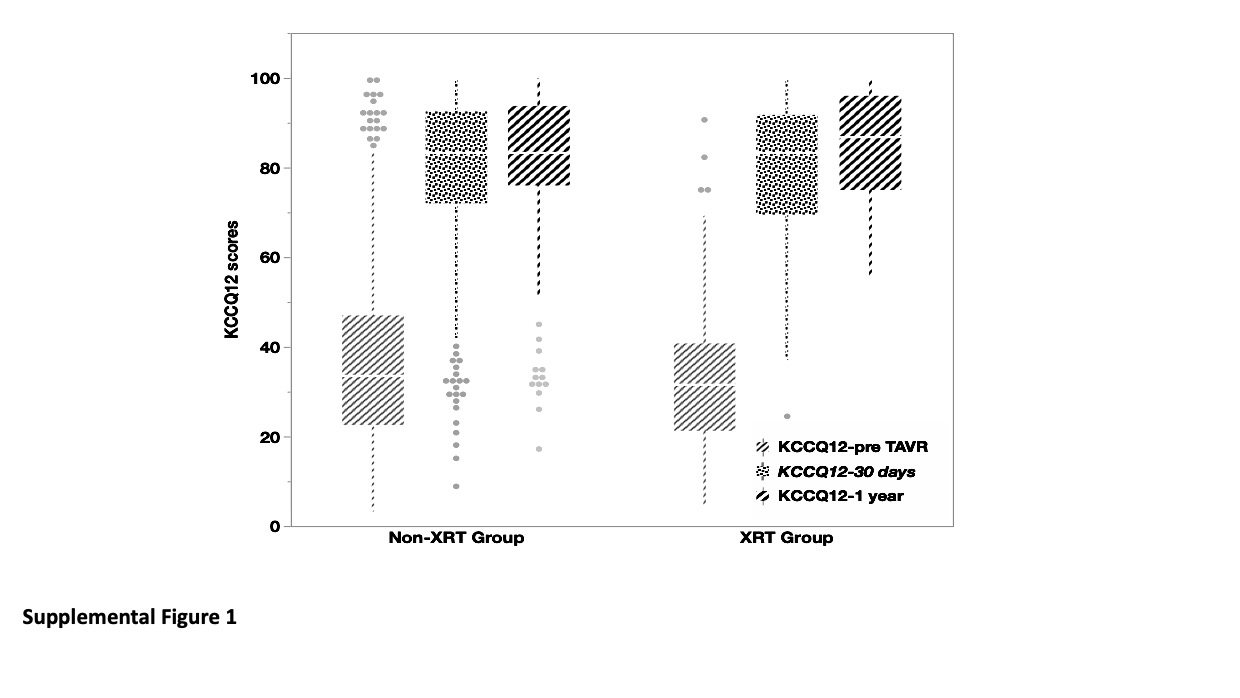

Supplement: Supplementary file 1 — Figure S1. Graph showing KCCQ12 score at pre TAVR, 1 month and one-year post TAVR in the entire study population and separated into 2 subgroups: chest radiotherapy (XRT group) versus comparison (Non-XRT group). (JPG 79 kb) [file 40959_2019_44_MOESM1_ESM.jpg]

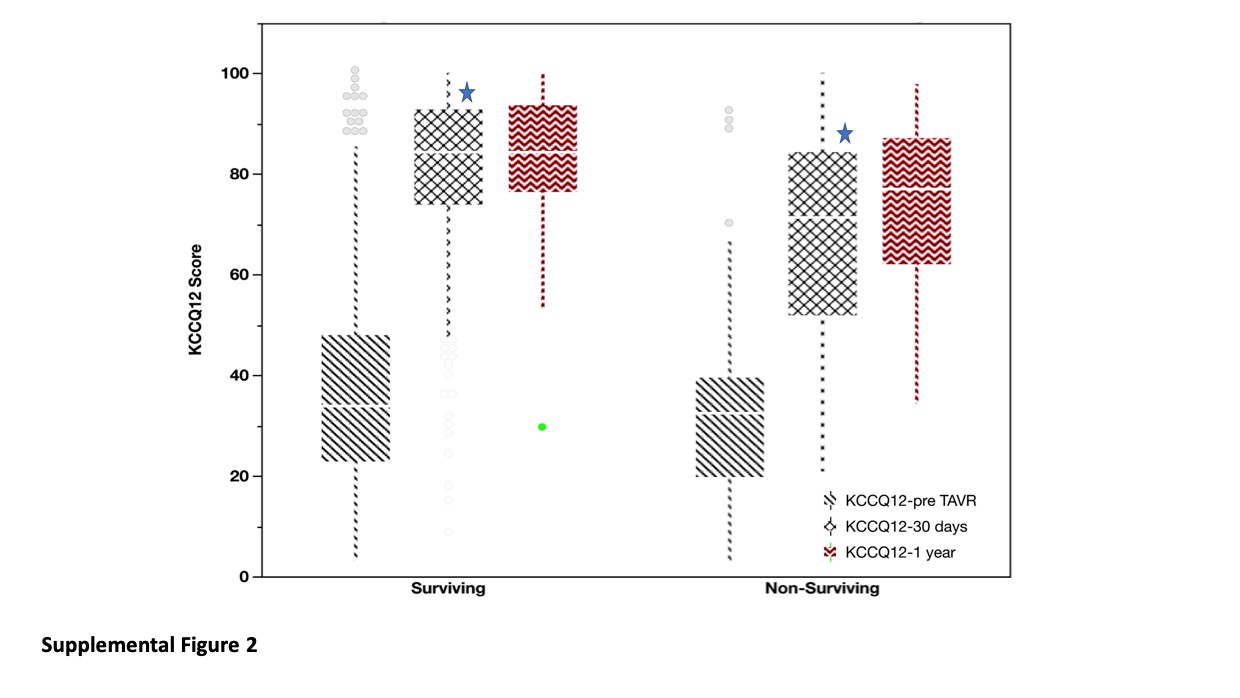

Supplement: Supplementary file 2 — Figure S2. Graph showing KCCQ12 score at pre TAVR, 1 month and one-year post TAVR in the entire study population and separated into 2 subgroups: surviving vs non-surviving groups). * denotes statistically significant differences among the groups. (JPG 87 kb) [file 40959_2019_44_MOESM2_ESM.jpg]
