# Supplementary material for: Clinical outcomes after transcatheter aortic valve replacement in cancer survivors treated with ionizing radiation
Source: Cardiooncology. 2019 Jul 22;5:8. doi: 10.1186/s40959-019-0044-7 (PMC6897372; doi:10.1186/s40959-019-0044-7)
Supplement: Supplementary file 3 — Table S1. Clinical Outcomes in Relation to Types of Malignancies Treated with Ionizing Radiation. (DOCX 16 kb) [file 40959_2019_44_MOESM3_ESM.docx]

**Additional file 3: Table S1. Clinical Outcomes in Relation to Types of Malignancies Treated with Ionizing Radiation**

| Outcomes* | Breast Cancer (n=33) | Hodgkin's Lymphoma (n=23) | Lung Cancer (n=11) | *Others (n=8)* |
| --- | --- | --- | --- | --- |
| Early Events | | | | |
| ICU LOS (hrs.) | 43.67±39.41 | 47.08±57.63 | 46.2±32.17 | 39.82±24.96 |
| Hospital LOS (days) | 4.51± 3.34 | 4.48±4.17 | 5.45±4.25 | 3.375±2.77 |
| In Hospital AMI | 0 (0) | 0 (0) | 0 (0) | 0 (0) |
| In Hospital AF | 14 (42.4) | 11 (47.8) | 7 (63.6) | 4 (50) |
| In Hospital stroke | 1 (3) | 3 (13) | 0 (0) | 0 (0) |
| In Hospital cardiac arrest | 2 (6) | 1 (4.3) | 2 (18.2) | 0 (0) |
| In Hospital Mortality | 2(6) | 1 (4.3) | 2 (18.2) | 0 (0) |
| 30-day Mortality | 2 (6) | 1 (4.3) | 2 (18.2) | 0 (0) |
| Major bleed | 6 (18.1) | 4 (17.4) | 0 (0) | 1 (12.5) |
| Long-term Events | | | | |
| AMI and/or urgent PCI | 2 (6) | 2 (8.7) | 0 (0) | 0 (0) |
| Stroke/TIA | 4(12.1) | 5 (21.7) | 0 (0) | 0 (0) |
| HF admission | 11 (33.3) | 7 (30.4) | 2 (18.2) | 3 (37.5) |
| PPM implants | 9(27.2) | 2 (8.7) | 2 (18.2) | 2 (25) |
| CV Mortality | 3 (9.1) | 4 (17.4) | 4 (36.4) | 3 (37.5) |
| All-Cause Mortality | 5 (15.1) | 8 (34.7) | 5 (45.4) | 4 (50) |
| MACE | 20 (60.6) | 18 (78.2) | 6 (54.5) | 6 (75) |
| Quality of Life measures | | | | |
| KCCQ12 at 30 days | 79.89±17.57 | 77.26±19.47 | 73.03±20.53 | 87.57±15.5 |
| KCCQ12 at 1 year | 87.08±12.38 | 79.86±13.69 | 81.65±12.29 | 85.73±13.41 |

*Values are expressed as number (percentage) or mean ±SD. XRT indicates mediastinal radiation therapy; ICU-intensive care unit; LOS-length of stay; AF-atrial Fibrillation; AMI-acute myocardial infarction; PCI-percutaneous coronary intervention; TIA-transient ischemic attack; HF-heart failure; PPM-permanent pacemaker; CV-cardiovascular; MACE-major cardiovascular adverse event (CV death/MI/Stroke/HF); KCCQ12-Kansas City Cardiomyopathy Questionnaire.
